# The Involvement of Thiamine Uptake in the Virulence of *Edwardsiella piscicida*

**DOI:** 10.3390/pathogens11040464

**Published:** 2022-04-13

**Authors:** Xin Liu, Xinhui Wang, Boguang Sun, Li Sun

**Affiliations:** 1 CAS and Shandong Province Key Laboratory of Experimental Marine Biology, Institute of Oceanology, CAS Center for Ocean Mega-Science, Chinese Academy of Sciences, Qingdao 266071, China; liuxin1@qdio.ac.cn (X.L.); wangxinhui@qdio.ac.cn (X.W.); 2 Laboratory for Marine Biology and Biotechnology, Pilot National Laboratory for Marine Science and Technology, Qingdao 266003, China; 3 College of Earth and Planetary Sciences, University of Chinese Academy of Sciences, Beijing 100049, China

**Keywords:** *Edwardsiella piscicida*, thiamine, transporter, c-di-GMP, virulence

## Abstract

*Edwardsiella piscicida* is a pathogenic bacterium, which can infect a number of fish species and cause a disease termed edwardsiellosis, threatening global fish farming with high prevalence and mortality. Thiamine (Vitamin B1), functioning in the form of thiamine pyrophosphate (TPP), is essential for almost all organisms. Bacteria acquire TPP by biosynthesis or by transportation of exogenous thiamine. TPP availability has been associated with bacterial pathogenicity, but the underlying mechanisms remain to be discovered. The role of thiamine in the pathogenicity of *E. piscicida* is unknown. In this study, we characterized a thiamine transporter (TT) operon in *E. piscicida*. The deletion of the TT operon resulted in an intracellular TPP lacking situation, which led to attenuated overall pathogenicity, impaired abilities associated with motility and host cell adhesion, as well as decreased expression of certain flagellar and adhesion genes. Moreover, TPP starvation led to intracellular c-di-GMP reduction, and introducing into the TPP-suppressed mutant strain an exogenous diguanylate cyclase for c-di-GMP synthesis restored the virulence loss. Taken together, this work reveals the involvement of thiamine uptake in the virulence regulation of *E. piscicida*, with c-di-GMP implicated in the process. These finding could be employed to explore potential drug targets against *E. piscicida*.

## 1. Introduction

*Edwardsiella piscicida* is a Gram-negative, facultatively anaerobic bacterium. The genus *Edwardsiella* is currently composed of five species, including *E. piscicida*, *E. ictulari*, *E. anguillarum*, *E. tarda*, and *E. hoshinae* [1]. *E. piscicida* is a relatively new species that contains the pathogenic fish isolates formerly considered to be *E. tarda* [2]. *E. piscicida* was isolated from freshwater, estuary, marine environments, and animal tissues. It can infect a wide range of animals, including fish, reptiles, birds, and mammals [1,3]. It is a major threat to global fish farming by causing edwardsiellosis, a severe disease featured by ascites, hernia, exophthalmia, and severe lesions of internal organs in numerous fish species, such as channel catfish (*Ictalurus punctatus*), common carp (*Cyprinus carpio*), Japanese eel (*Anguilla japonica*), Japanese flounder (*Paralichthys olivaceus*), largemouth bass (*Micropterus salmoides*), striped bass (*Morone saxatilis*), and turbot (*Scophthalmus maximus*) [3,4].

The known virulence factors in *E. piscicida* include flagella, adhesion factors such as adhesins and invasins, serum resistance proteins, and the type III and type VI secretion systems [5,6,7,8,9,10,11,12,13]. During infection, *E. piscicida* is motivated by the rotation of flagella in response to host cues and attaches to the entry sites on the host, such as the skin, gastrointestine, and gills, via adhesion factors [14,15]. The pathogen then penetrates the tissue barrier, replicates inside host cells including phagocytes, and sabotages host immune responses with an armory of virulence factors and their mediators [4,14,16,17].

Thiamine is an essential microelement required for almost all organisms [18,19]. Its active form, thiamine pyrophosphate (TPP), participates in various biological processes by acting as a coenzyme or through non-coenzyme mechanisms [18,20]. Bacteria acquire thiamine and TPP by de novo synthesis and/or by uptake via transporters. Bacterial transporters of thiamine can be classified as ABC-type transporters, energy coupling factor (ECF) importers, NiaP transporters, and PnuT transporters [21]. The *Salmonella thiBPQ* operon encodes a representative ABC-type thiamine uptake system, which comprises a periplasmic thiamine binding protein, a transmembrane channel protein, and an ATPase, together forming an ATP-binding cassette-type transporter [22,23,24]. Two reports indicated an association of thiamine transport with bacterial pathogenicity. In these reports, the mutation of a thiamine transporter in *Listeria monocytogenes* and *Verticillium dahliae* resulted in reduced bacterial virulence [25,26]. Otherwise, the role of thiamine uptake in the pathogenicity of bacteria, including *E. piscicida*, remains to be investigated.

In carbohydrate metabolism, TPP functions as a cofactor for enzymes such as transketolase, α-ketoacid decarboxylase, α-ketoacid dehydrogenase, and acetolactate synthase [20]. Transketolase is a key enzyme in the pentose phosphate pathway, which in bacteria provides the pentose skeleton for the biosynthesis of cyclic-di-GMP (c-di-GMP) [27]. C-di-GMP is a second messenger that mediates many aspects of bacterial lifecycle, such as motility, biofilm formation, replication, and virulence [28,29]. Bacterial c-di-GMP is synthesized by diguanylate cyclases (DGCs) and degraded by c-di-GMP-specific phosphodiesterases (PDEs) [30]. The concentration dynamic of c-di-GMP is controlled by an array of DGCs and PDEs upon intracellular and/or environmental cues, and subsequently converted to physiological responses via various c-di-GMP binding effectors, including RNA and proteins [29]. The RNA effectors refer to Class I and Class II c-di-GMP riboswitches, which control the downstream gene transcription and translation, respectively [31]. The known protein receptors for c-di-GMP include transcriptional regulators, PilZ proteins, YajQ proteins, and ATPases [29]. These effectors connect c-di-GMP signaling to gene expression and protein functionality, and interplay with other molecular pathways such as quorum sensing [32]. In pathogenic bacteria, a number of virulence genes and traits are regulated by c-di-GMP signaling in a dynamic and varied fashion. In general, low levels of c-di-GMP seem to be programmed for acute infection and associated with the bacterial ability to destroy or penetrate host cells [29]. In contrast, high levels of c-di-GMP seem to favor bacterial colonization of host tissues [29]. The genome of *E. piscicida* contains at least 11 c-di-GMP associated genes, encoding DGCs, PDEs, and an effector protein [33]. However, neither c-di-GMP signaling nor its possible role in the pathogenicity of *E. piscicida* has been reported thus far.

In this work, we characterized a *thiBPQ*-type thiamine transporter operon in *E. piscicida* and investigated its contribution to pathogenicity. We then identified c-di-GMP as a mediating molecule underlying the virulence regulation of thiamine uptake. Our results suggest that thiamine acquisition could be targeted for the development of new therapies against *E. piscicida*.

## 2. Results

### 2.1. E. piscicida Thiamine Transporter (TT) Operon Is Required for In Vivo Dissemination of E. piscicida

*E. piscicida* ZW1 possesses a *thiBPQ t*hiamine transporter operon, which is 100% identical to that of *E. piscicida* EIB202. The sequences of *thiB* (ETAE_0610), *thiP* (ETAE_0609), and *thiQ* (ETAE_0608) overlap and form the coding part of the operon. The deduced amino acid sequences of ThiB, ThiP, and ThiQ are 331, 535, and 237 residues in length, and possess the feature domains of bacterial extracellular solute-binding protein (Pfam ID: PF01547), binding-protein-dependent transport system inner membrane component (Pfam ID: PF00528), and ATP-binding cassette transporter (Pfam ID: PF00005), respectively (Supplementary Figure S1). ThiB, ThiP, and ThiQ share 72.51% to 68.88%, 76.82% to 71.06%, and 68.49% to 60.85% sequence identities, respectively, with their homologues in *Salmonella enterica*, *Escherichia coli*, *Hafnia alvei*, *Klebsiella pneumoniae*, *Serratia liquefaciens*, and *Yersinia kristensenii* (Supplementary Figure S1). The *thiBPQ* operon was named TT for short.

**Figure 1:**
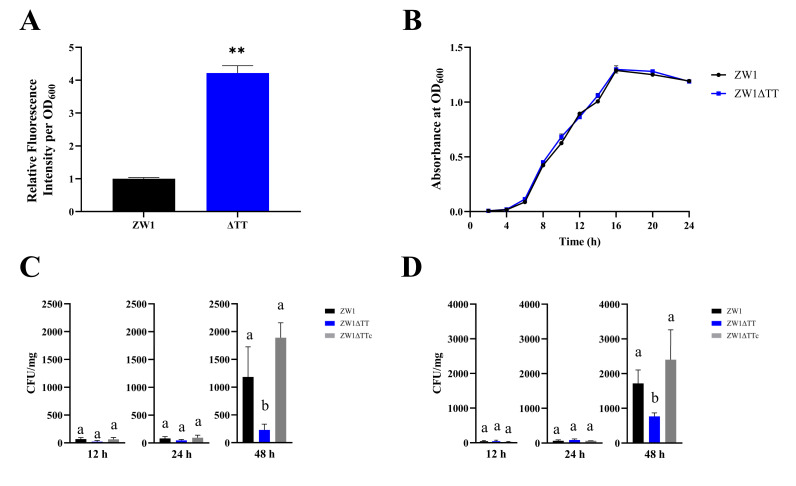
The effects of *thiBPQ* knockout on the TPP content, growth, and infection of ZW1. (**A**) The intracellular TPP levels of ZW1 and ZW1ΔTT were analyzed with a reporter plasmid. The higher the fluorescence intensity, the lower the TPP content. (**B**) Growth curves of ZW1 and ZW1ΔTT in Luria–Bertani broth (LB) medium. (**C**,**D**) Turbot were infected with ZW1, ZW1ΔTT, or ZW1ΔTTc, and bacterial recoveries from spleen (**C**) and kidney (**D**) were determined by plate count at different time points. Data are presented as means ± SD, *n* = 3. ** *p* < 0.01. Data marked with different letters have statistically significant differences (*p* < 0.05).

To inspect the functional importance of the TT operon, an isogenic mutant of ZW1, ZW1ΔTT, was constructed, in which almost the entire TT operon, from the 28th amino acid residue of ThiB to the 205th residue of ThiQ, was deleted markerlessly. The thiamine level in ZW1ΔTT was analyzed using a previously verified thiamine sensing reporter plasmid [34]. Compared to ZW1, ZW1ΔTT produced significantly enhanced GFP fluorescence signal, implying a relative lack of TPP in ZW1ΔTT (Figure 1A). This result indicates that the TT operon is essential to thiamine transport. Growth analysis showed that ZW1ΔTT exhibited a growth curve similar to that of ZW1 (Figure 1B). In vivo infection showed that following inoculation into turbot, the numbers of ZW1ΔTT in the spleen and kidney of the fish were similar to that of ZW1 at 12 and 24 hpi, but were significantly lower than that of ZW1, as well as the TT complement strain ZW1ΔTTc at 48 hpi (Figure 1C,D). In addition, the mortality-inducing capacities of ZW1ΔTT and ZW1 were also compared. The results showed that at the same dose of infection, both ZW1ΔTT and ZW1 caused 100% mortality; however, ZW1ΔTT-induced death was much delayed than that induced by ZW1 (Supplementary Figure S2). These results indicate that thiamine uptake is required for the pathogenicity of *E. piscicida*.

### 2.2. TT Knockout Reduces E. piscicida Motility and Flagellar Gene Expression

**Figure 2:**
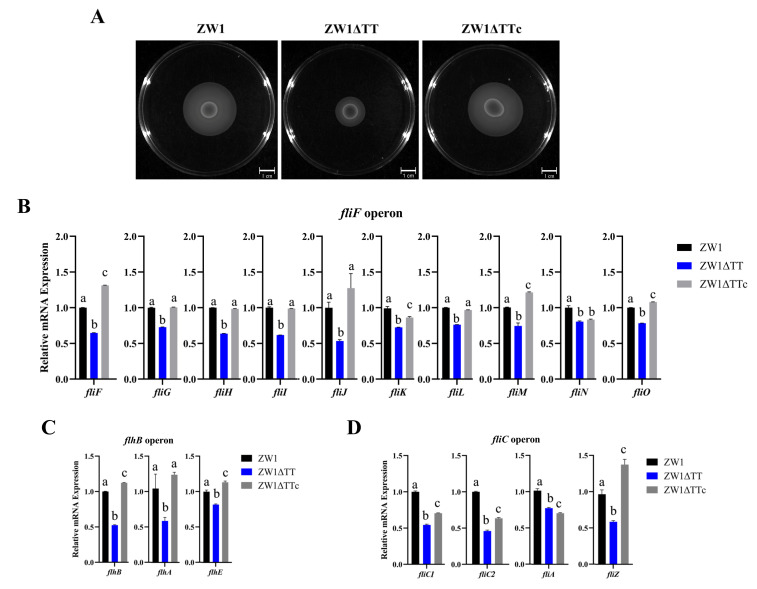
The effects of TT knockout on bacterial motility and flagellar gene expression. (**A**) Swimming abilities of ZW1, ZW1ΔTT, and ZW1ΔTTc were compared using LB medium containing 0.3% (w/v) agar. (**B**–**D**) The expression of flagellar genes of the *fliF* (**B**), *flhB* (**C**), and *fliC* (**D**) operons in ZW1, ZW1ΔTT, and ZW1ΔTTc were measured by qRT-PCR with *topA* as an internal reference. For the convenience of comparison, the expression levels in ZW1 were normalized as 1. Data are presented as means ± SD, *n* = 3. Data marked with different letters have statistically significant differences (*p* < 0.05).

Soft agar swimming assay showed that ZW1ΔTT displayed apparently reduced motility in comparison to ZW1 or ZW1ΔTTc (Figure 2A, Supplementary Figure S3). Since flagella play an important role in motility, the expressions of *flhB*, *flhD*, *flgA*, *flgB*, *fliC1*, *fliD*, and *fliF*, the first genes of 7 flagellar operons, were analyzed by qRT-PCR. The results showed that the expressions of *fliF*, *flhB,* and *fliC1* in ZW1ΔTT were significantly repressed compared to those in ZW1 or ZW1ΔTTc (Figure 2B–D, Supplementary Figure S4). Moreover, the expressions of the other genes in the *fliF* operon (*fliG*, *fliH*, *fliI*, *fliJ*, *fliK*, *fliL*, *fliM*, *fliN*, and *fliO*), the *flhB* operon (*flhA* and *flhE*), and the *fliC* operon (*fliC2*, *fliA*, and *fliZ*) were also significantly reduced in ZW1ΔTT (Figure 2B–D). These results demonstrate a positive effect of thiamine uptake on the motility of *E. piscicida*, which may be at least partially through the transcriptional regulation of the flagellar genes.

### 2.3. TT Knockout Impairs E. piscicida Ability to Attach Host Cells

**Figure 3:**
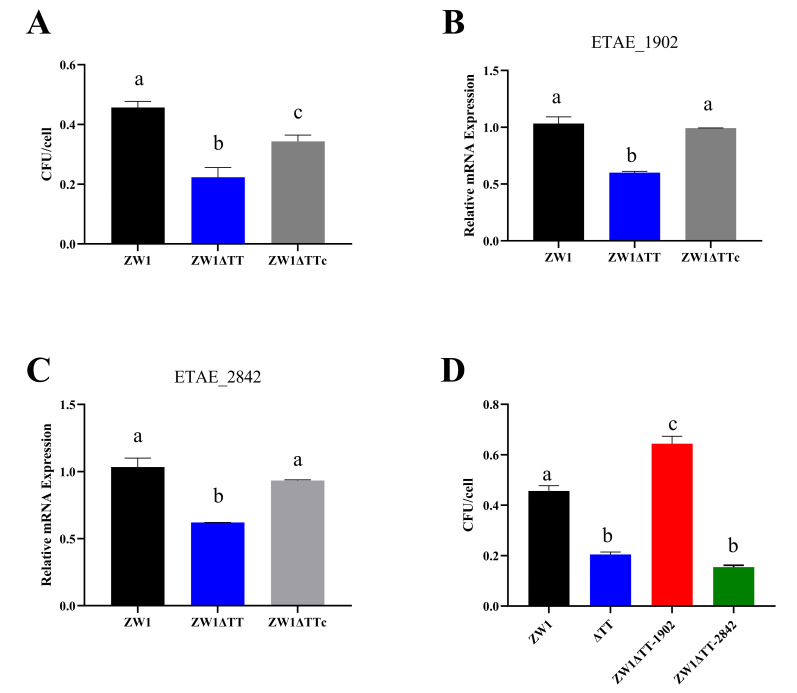
The effects of TT knockout on bacterial adhesion to fish cells and expression of adhesin and invasin genes. (**A**) Turbot peripheral blood leukocytes (PBLs) were incubated with ZW1, ZW1ΔTT, or ZW1ΔTTc for 2 h, and the number of the cell-attached bacteria was determined. (**B**,**C**) The expression of the adhesin/invasin genes ETAT_1902 (**B**) and ETAE_2842 (**C**) in ZW1, ZW1ΔTT, and ZW1ΔTTc were measured by qRT-PCR with *topA* as an internal reference. For the convenience of comparison, the expression levels in ZW1 were normalized as 1. (**D**) Turbot PBLs were incubated with ZW1, ZW1ΔTT, or ZW1ΔTT overexpressing ETAE_1902 or ETAT_2842 for 2 h, and the number of the cell-attached bacteria was determined. Data are presented as means ± SD, *n* = 3. Data marked with different letters have statistically significant differences (*p* < 0.05).

Cellular infection study showed that when turbot peripheral blood leukocytes (PBLs) were infected with ZW1, ZW1ΔTT, or ZW1ΔTTc for 2 h at an MOI of 1:1, the majority of the bacteria were still at the stage of cellular attachment (Supplementary Figure S5). Compared to ZW1, ZW1ΔTT exhibited a significantly decreased ability to attach to PBLs (Figure 3A). qRT-PCR analysis of the expression of eight adhesion factor genes showed that the mRNA levels of two genes, i.e., ETAE_1902 and ETAE_2842, were significantly lower in ZW1ΔTT than in ZW1 or ZW1ΔTTc (Figure 3B,C, Supplementary Figure S6). Furthermore, overexpression of ETAE_1902 in ZW1ΔTT significantly increased bacterial adhesion to PBLs to the extent comparable to that of ZW1 (Figure 3D). These results indicate that thiamine uptake participates in bacterial attachment to host cells in a manner that involves the transcriptional regulation of the adhesin gene ETAE_1902.

### 2.4. C-di-GMP Is Involved in the Thiamine Uptake-Associated Virulence Regulation of E. piscicida

**Figure 4:**
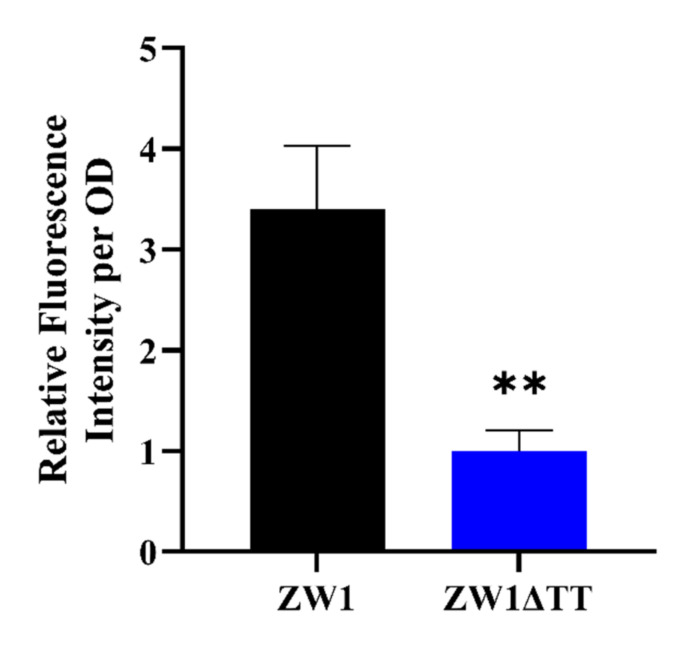
The effect of TT knockout on c-di-GMP level. The intracellular c-di-GMP content in ZW1 and ZW1ΔTT was analyzed with a reporter plasmid. The lower the fluorescence intensity, the lower the c-di-GMP content. Data are presented as means ± SD, *n* = 3. ** *p* < 0.01.

**Figure 5:**
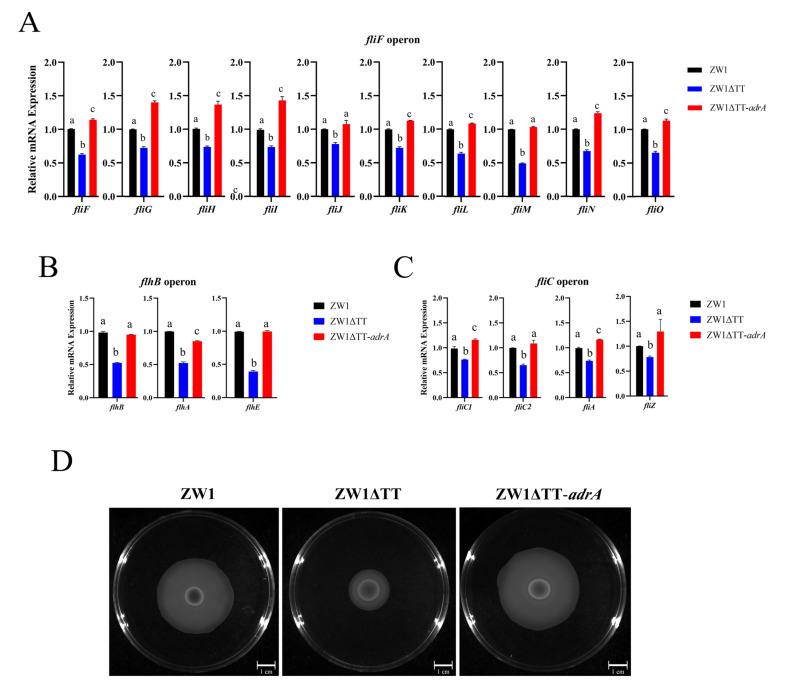
Thiamine uptake affects flagellar gene expression and bacterial motility through c-di-GMP. The gene expression of the *fliF* (**A**), *flhB* (**B**), and *fliC* (**C**) operons in ZW1, ZW1ΔTT, and ZW1ΔTT-*adrA* was measured by qRT-PCR with *topA* as an internal reference. For the convenience of comparison, the expression levels in ZW1 were normalized as 1. Data are presented as means ± SD, *n* = 3. Data marked with different letters have statistically significant differences (*p* < 0.05). (**D**) The motility of ZW1, ZW1ΔTT, and ZW1ΔTT-*adrA* on LB medium containing 0.3% (w/v) agar.

**Figure 6:**
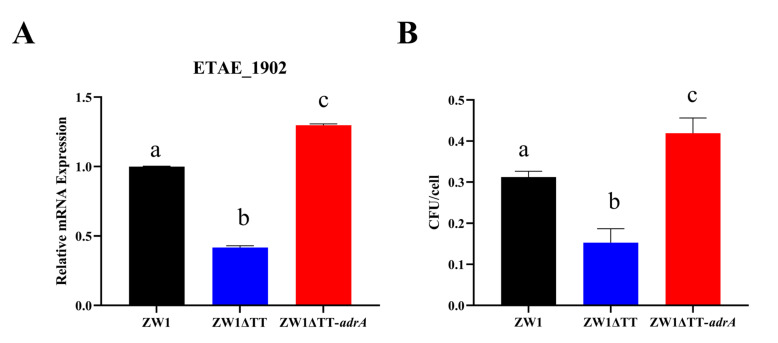
Thiamine uptake affects ETAE_1902 gene expression and bacterial adhesion through c-di-GMP. (**A**) The expression of ETAE_1902 in ZW1, ZW1ΔTT, and ZW1ΔTT-*adrA* was measured by qRT-PCR with *topA* as an internal reference. For the convenience of comparison, the expression levels in ZW1 were normalized as 1. (**B**) Turbot PBLs were incubated with ZW1, ZW1ΔTT, or ZW1ΔTT-*adrA* for 2 h, and the number of cell-attached bacteria was determined. Data are presented as means ± SD, *n* = 3. Data marked with different letters have statistically significant differences (*p* < 0.05).

The effect of TT knockout on the intracellular level of c-di-GMP was examined with a c-di-GMP sensing reporter plasmid [35]. Compared to ZW1, ZW1ΔTT displayed a significantly more intense fluorescence signal, indicating a lower amount of c-di-GMP in ZW1ΔTT (Figure 4). To assess the importance of c-di-GMP to the virulence associated traits of ZW1, the strain ZW1ΔTT-*adrA* was constructed, which is ZW1ΔTT overexpressing *adrA*, a DGC gene with certified function in c-di-GMP synthesis [36]. qRT-PCR showed that compared to ZW1ΔTT, the expressions of the *fliF*, *flhB,* and *fliC* operons and the adhesin gene ETAE_1902 were all significantly increased in ZW1ΔTT-*adrA* to the levels comparable to those in ZW1 (Figure 5A–C and Figure 6A). Consequently, the motility and host cell adhesion of ZW1ΔTT-*adrA* were also increased (Figure 5D, Supplementary Figure S7, Figure 6B). Together these results suggest that thiamine uptake likely contributes to the pathogenicity of *E. piscicida* through c-di-GMP.

## 3. Discussion

In this study we investigated the involvement of thiamine uptake in the virulence of *E. piscicida*. Thiamine is the precursor of TPP, which is an important coenzyme in central carbon metabolism and linked to virulence gene expression in intracellular bacteria [22,37]. In *Xanthomonas oryzae*, one of the TPP synthesis gene, *thiG*, was shown to be required for the full virulence of the bacterium [38]. In *Pseudomonas aeruginosa*, the deletion of *thiL*, which encodes an enzyme catalyzing the phosphorylation of thiamine monophosphate to produce TPP, resulted in markedly attenuated pathogenicity [39]. In *V. dahliae*, a thiamine transporter protein, VdThit, was important for bacterial invasion [25]. In *L. monocytogenes*, both thiamine uptake and the synthesis of thiamine precursors were essential to intracellular replication [26]. In *E. piscicida*, the genes for thiamine uptake and synthesis are present in the genome, however, their roles in pathogenicity are unknown. In this study, we found that deletion of the *thiBPQ* thiamine transporter operon led to a markedly reduced intracellular level of TPP, indicating that *E. piscicida* acquires thiamine mainly through transportation from exogenous sources. Further, we discovered that the virulence of *E. piscicida* was significantly attenuated under the condition of reduced TPP. These results, together with the previous observations, suggest that thiamine, either by exogenous uptake or by biosynthesis, is possibly universally required for bacterial pathogenicity. However, the underlying mechanism remains to be explored.

In this study, we found that the swimming and host cell attachment abilities were obviously impaired in ZW1ΔTT. The genes whose expression may be responsible for the difference in these abilities were identified. For motility, they were *fliF-O*, *flhBAE*, and *fliC1C2AZ*, most of which are known to encode the structure proteins of the flagellar apparatus [40,41]. Notably, the *fliC* genes encoding flagellins, the flagellar filament structural proteins, have been reported to be essential for the virulence of *E. piscicida* [5]. For the genes associated with cellular adhesion, ETAE_1902 was found to be significantly repressed in expression upon TPP suppression. ETAE_1902 encodes a YadA-type adhesin. YadA is an autotransporter adhesin and a major virulence factor of *Y. enterocolitica* in which YadA mediates a bacterial attachment to the extracellular matrix components of eukaryotic host cells [42]. In our work, overexpression of ETAE_1902 restored the lost adhesion capacity of ZW1ΔTT, suggesting that YadA was likely a major thiamine-regulated factor in *E. piscicida* for host attachment.

**Figure 7:**
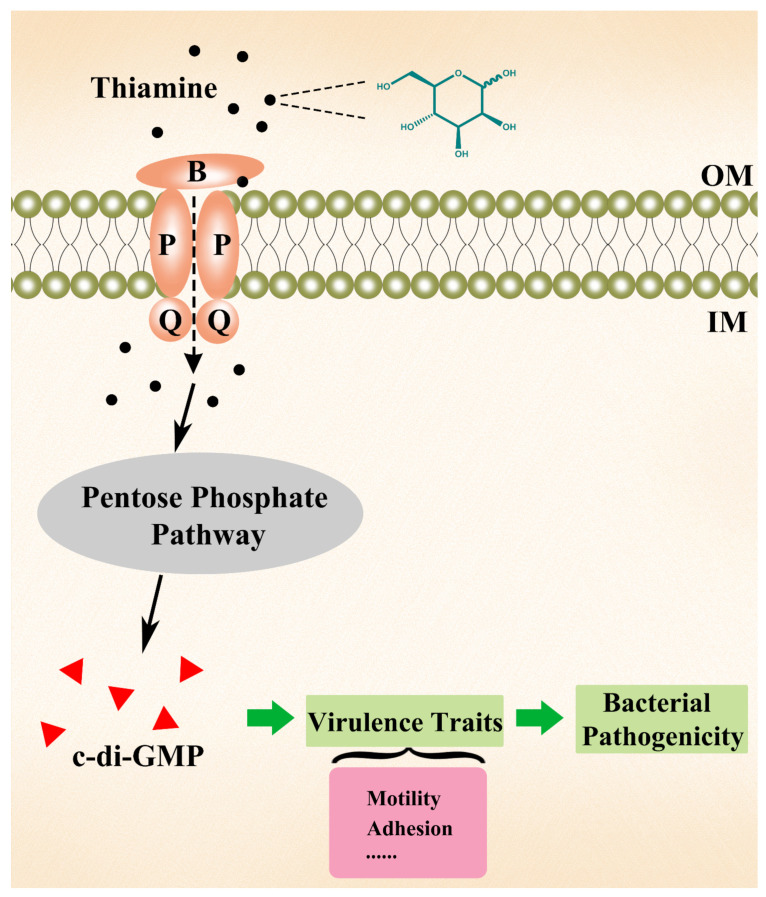
Schematic diagram of the possible mechanism underlying the effects of thiamine uptake on *Edwardsiella piscicida* pathogenicity. Exogenous thiamine enters bacterial cells through the thiamine transporter and fuels the pentose phosphate pathway for the biosynthesis of c-di-GMP, which regulates the expression of virulence genes and consequently affects the virulence traits, such as motility and adhesion, leading eventually to altered bacterial pathogenicity.

In the present study, we conducted a search for the possible molecules and signaling routes linking thiamine availability to virulence traits in *E. piscicida*. We found that c-di-GMP synthesis was significantly repressed in the TPP-scarce mutant, thereby supporting the possibility that TPP might facilitate the biosynthesis of cyclic nucleotide second messengers by promoting the pentose phosphate pathway through transketolase [27]. C-di-GMP is known to play a role in several aspects of bacterial infection, including host cell adherence and invasion, bacterial motility, and the expression of virulence-associated genes [29,43]. For instance, in *L. monocytogenes* and *S. enterica*, the cellular invasion capacities are dampened by high concentrations of c-di-GMP [44,45] In *Clostridium difficile*, the expression of the exotoxin genes *tcdA* and *tcdB* were repressed by c-di-GMP through a riboswitch-gated transcriptional activator [46]. In *P. aeruginosa*, the *cdrA* gene encoding a key adhesin in the exopolysaccharides (EPS), which participate in host cell adhesion and biofilm formation, was transcriptionally induced in response to c-di-GMP elevation [29,47]. In *Borrelia burgdorferi*, the deletion of the gene coding for a c-di-GMP effector, PliZ, resulted in reduced bacterial motility and virulence [48]. In this work, we showed that c-di-GMP elevation induced by an exogenous DGC enzyme could restore the lost motility and host adhesion, as well as the associated gene expression, caused by TPP undersupply, therefore demonstrating the importance of c-di-GMP in the regulation of virulence associated with thiamine availability in *E. piscicida*. The possible mechanism underlying these observations may be as follows: the bacteria acquire exogenous thiamine via transportation to fuel the pentose phosphate pathway for the biosynthesis of c-di-GMP, which regulates virulence gene expression through effectors yet to be discovered and consequently affects the infective capacity of the bacteria, such as motility, host cell attachment, and presumably others, and eventually alters the bacterial pathogenicity (Figure 7).

## 4. Materials and Methods

### 4.1. Bacterial Strains and Growth Conditions

*E. piscicida* ZW1 was isolated from a diseased turbot from ZhuWang, Yantai, Shandong province, China in 2019. *E. coli* strains were from American Type Culture Collection (ATCC). All strains were grown in Luria–Bertani broth (LB) medium at 28 °C (for *E. piscicida*) or 37 °C (for *E. coli*). Where appropriate, polymyxin B, ampicillin, kanamycin, and chloramphenicol were supplemented at the concentrations of 50, 100, 50, and 30 µg/mL, respectively. The bacterial strains are listed in Supplementary Table S1.

### 4.2. Gene Deletion, Complementation, and Overexpression

For *thiBPQ* deletion, pDM4 was used for in-frame deletion as described previously [49]. In brief, fragments upstream and downstream of the *thiBPQ* operon were amplified and overlapped by PCR and then inserted to pDM4 at the indicated endonuclease sites (Table 1). The deletion mutant, ZW1ΔTT, was generated by two-step homologous recombination and verified by PCR and sequencing.

For the construction of the TT complement strain, the plasmid pCP1 was created by adding a MCS linker to pwtCas9-bacterial (Addgene 44250) at between the *Afl*II and *Xho*I sites. The entire *thiBPQ* with the promoter region was amplified by PCR and inserted into pCP1 at the indicated sites (Table 1). The resulting plasmid was introduced into ZW1ΔTT by electroporation, yielding the complement strain ZW1ΔTTc.

For gene overexpression, the PCR products of the indicated genes (adhesin/invasin genes from *E. piscicida* ZW1; *adrA* from *E. coli* MG1655) were inserted into pwtCas9-bacterial between the *Bgl*II and *Xho*I sites with the ClonExpress^®^ II One Step Cloning Kit (Vazyme, Nanjing, China) to enable an inducible expression of these genes under a tetracycline promoter. For invasin/adhesin overexpression, the control plasmid pOE1c was constructed by blunting and ligating the *Bgl*II and *Xho*I digestion product of pwtCas9-bacterial. For *adrA* overexpression, the control plasmid pOE1-adrA* was constructed as reported previously [36], which expressed an inactivated AdrA. The resulting plasmids were introduced into indicated *E. piscicida* strains by electroporation. Where appropriate, the expression was induced by adding 1 μM anhydrotetracycline.

The TPP sensing plasmid pNmA was commercially synthesized with pEGFP-N2 as the bone vector. The plasmid was exactly the same as described in a previous publication [34], which contains a natural TPP riboswitch of serogroup A *Neisseria meningitidis*. In this plasmid, the TPP riboswitch forms a stem–loop structure in the presence of TPP, resembling a Rho-independent transcription terminator, thereby preventing transcription of the downstream *gfp* gene. pEGFP-N2 was used as the control plasmid. The plasmids were introduced into indicated *E. piscicida* strains by electroporation.

The c-di-GMP sensing plasmid pBc3–5 was commercially synthesized with pEGFP-N2 as the bone vector. This plasmid put a well-characterized c-di-GMP riboswitch [35] between a synthetic constitutive promoter J23100 and a strong RBS B0034, both were functionally verified and have frequently been documented as a combination for driving the expression of various genes such as *gfp* (BBa_K3740058 and others). The details and updates can be found at http://parts.igem.org (accessed on 2 March 2022). The above riboswitch-containing expression cassette was placed at the *Sma*I site of pEGFP-N2 and before the *gfp* gene. In the absence of c-di-GMP, the riboswitch terminates the transcription of the downstream *gfp* gene. pEGFP-N2 was used as the control plasmid. The plasmids were introduced into indicated *E. piscicida* strains by electroporation.

All primers are listed in Table 1.

### 4.3. Experimental Animals

Healthy *Scophthalmus maximus* (average 20 g or 500 g) were purchased from Hai-Shuo-Jia-Yuan aquaculture company in Qingdao city, Shandong province, China. The fish were maintained at 19 °C in aerated seawater and acclimatized in the laboratory for 2 weeks prior to experiments. Before experiments, fish were verified to be healthy by examination of potential bacterial existence in kidney and spleen as described previously [51].

### 4.4. In Vivo Infection

To compare the in vivo infectivity of *E. piscicida* ZW1, ZW1ΔTT, and ZW1ΔTTc, the abilities of these strains to disseminate in host tissues were examined. For this purpose, ZW1, ZW1ΔTT, and ZW1ΔTTc were cultured as above and collected at OD_600_ 0.5. The bacteria were washed with and resuspended in PBS to a concentration of 2 × 10^6^ CFU/mL. Turbot (average 20 g) were randomly divided into three groups (9 fish/group). The fish of Group 1 (control group) were intramuscularly injected with 100 μL ZW1. The fish of Groups 2 and 3 were similarly injected with 100 μL ZW1ΔTT and ZW1ΔTTc, respectively. The dose of injection in all groups was 2 × 10^5^ CFU/fish. At 12 h, 24 h, and 48 h post-infection, fish were euthanized with an overdose of tricaine methanesulfonate (Sigma). The spleen and kidney were collected aseptically, homogenized, and serially diluted in PBS. The diluted homogenates were plated on LB agar and incubated at 28 °C for 24 h. The colonies were counted in number. To verify the identity of the bacteria, several clones were randomly picked for 16S rDNA sequencing by Tsingke, China. To examine the mortality-inducing capacity of the *E. piscicida* strains, turbot of two groups (10 fish/group) were injected intramuscularly with ZW1 or ZW1ΔTT as described above. The fish were monitored daily for survival for 7 days.

### 4.5. Soft Agar Swimming Assay

*E. piscicida* ZW1, ZW1ΔTT, and ZW1ΔTTc were cultured as above and collected at OD_600_ 0.5. The bacteria were washed with and resuspended in PBS to a concentration of 1 × 10^8^ CFU/mL. Ten-microliter of bacterial suspension was dropped onto LB plates containing 0.3% (w/v) agar. The plates were incubated at 28 ℃ for at least 2 days and observed for bacterial motility.

### 4.6. Cellular Adhesion Assay

Peripheral blood leukocytes (PBLs) were isolated from turbot (average 500 g) as reported previously [52] and adjusted to 10^7^ cell/mL in L-15 medium (Gibico, Grand Island, NY, USA). *E. piscicida* strains were prepared as described above and suspended in L-15. PBLs were infected with *E. piscicida* at an MOI of 1:1 and incubated at 22 °C for 2 h with constant slow rotation. The cells were then lysed with 1% Triton X-100, and the lysate was serially diluted and plated on LB agar plates. The plates were incubated at 28 °C, and the colonies were counted and verified as above. To calculate the amount of internalized bacteria, the experiment was conducted as described as above, except that the extracellular and cell surface-attached bacteria were killed by treating with gentamycin (200 μg/mL) for 1 h before plate count.

### 4.7. qRT-PCR

Bacteria were prepared as described above, and 10^9^ cells were lysed with Bacteria RNA Extraction Kit (Vazyme, Nanjing, China). RNA was extracted using Bacterial RNA Kit (Omega Bio-Tek, Guangzhou, China), with the optional on-membrane DNase I treatment step included to remove the residual genomic DNA. One microgram of purified RNA was used for reverse transcription with the RevertAid First Strand cDNA Synthesis Kit (Thermo Fisher Scientific, Waltham, MA, USA). qRT-PCR was performed with three biological samples using the QuantStudio 3 Real-Time PCR System. Relative transcription was quantified by the comparative Ct (2^−ΔΔCT^) method with *topA* as an internal control [50]. For the comparison of flagellar and adhesion gene expression in ZW1, ZW1ΔTT, and ZW1ΔTTc, the empty plasmid pCP1 was introduced into both ZW1 and ZW1ΔTT. For the comparison of flagellar and adhesion gene expression in ZW1, ZW1ΔTT, and ZW1ΔTT-*adrA*, the control plasmid pOE1-adrA* expressing an inactivated AdrA was introduced into ZW1 and ZW1ΔTT. In all cases, the gene expression level in ZW1 was set as 1.

All primers are listed in Table 1.

### 4.8. Fluorescence Intensity Quantification

Bacteria were prepared as above and resuspended in PBS. The suspension (200 μL) was transferred to a Costar^®^ 3603 plate. The visible light absorption at 600 nm and the fluorescence intensity (FI) (excitation and emission wavelengths at 485 nm and 515 nm, respectively) were measured with a BioTek Synergy H1 multifunction reader (Winooski, VT, USA). The relative FI was calculated as the FI value per OD_600_. The data were presented as the relative FI value of the reporter strain subtracted by the value of its control strain, which harbored the empty plasmid pEGFP-N2.

### 4.9. Statistical Analysis

All experiments were performed three times. Statistical analyses were carried out with the SPSS software (SPSS Inc., Chicago, IL, USA). Data were analyzed with Student’s t test or one-way ANOVA. Statistical significance was defined as *p* < 0.05.

## 5. Conclusions

In conclusion, the present work demonstrates that thiamine uptake abrogation imposed by deletion of the *thiBPQ*-type thiamine transporter is able to create an intracellular TPP-scarce situation, which attenuates the overall pathogenicity of *E. piscicida*, and reduces host colonization and the expression of the associated virulence genes. TPP starvation leads to intracellular c-di-GMP suppression, probably through the pentose phosphate pathway. C-di-GMP supplementation restored the impaired virulence-associated gene expression and phenotypes caused by TPP reduction. These findings reveal an important role of thiamine uptake and c-di-GMP in the pathogenicity of *E. piscicida* and the participation of TPP in c-di-GMP signaling. Furthermore, by indicating an essentialness of TPP in bacterial virulence, our results also suggest that the TPP/thiamine acquisition pathway might be considered as the target for anti-*E. piscicida* drugs or therapies. C-di-GMP signaling and its role in the regulation of *E. piscicida* virulence require further investigation.

## Figures and Tables

**Table 1:** Primers used in this study.

Primer	Sequence (5′-3′)	Source
TTF1	ATCTCTAGACCCTATTGAACAACGCCAGC (*Xba*I)	This study
TTR1	ATATGGCCGGTCAGAACGGGCTTGTCG	This study
TTF2	TTCTGACCGGCCATATCGCATACGACGG	This study
TTR2	GCTGTCGACGTACTCCAGCGTGAAGTCCA (*Sal*I)	This study
MCS linker-F	TTAAGTTAATTAACCTGCAGGGATATCAGATCTTCTAGACATATGGGATCCGAATTCCCATGGGTCGACAAGCTTGCGGCCGCC	This study
MCS linker-R	TCGAGGCGGCCGCAAGCTTGTCGACCCATGGGAATTCGGATCCCATATGTCTAGAAGATCTGATATCCCTGCAGGTTAATTAAC	This study
TTcF	TCCTCTAGACAAACGTCTTTCTAATCACGCACG (*Xba*I)	This study
TTcR	CCGAAGCTTCTATTCGGCCTGCTCTGGCA (*Hind*III)	This study
RT-*topA*-F	GCTATGAGGTAGAAGAGG	[50]
RT-*topA*-R	CCCATATACTTGCCAAAG	[50]
RT-1687-F	GCTATCACAGACGCTCTC	This study
RT-1687-R	GGCAATCATTCAGGATCAG	This study
RT-2842-F	AGATAGGACAATGGCTCAA	This study
RT-2842-R	CTGCGTAAATAGGGTCAAC	This study
RT-1902-F	TATAGCGTCGGTAAGTTCAA	This study
RT-1902-R	TGATGGGTAAAGCGGTAG	This study
RT-3032-F	GAGTCTGGCTATCACCTT	This study
RT-3032-R	TTATTGGCGGCATTATCG	This study
RT-2923-F	GATGACCGACCAGCATAA	This study
RT-2923-R	GTTGGCGTTCTGATAGTTG	This study
RT-0315-F	ATGTGGTTGATGGATGGA	This study
RT-0315-R	GTATTGGTGGCGGTAATG	This study
RT-0818-F	CAGGATGGTGTGATGATTG	This study
RT-0818-R	ACTTCCGTTGGCTATCTC	This study
RT-3034-F	ACCTGAGTGTCTGGAGTA	This study
RT-3034-R	CAGCGGCATCTTATTGAG	This study
RT-1987-F	TACGACTACGACGCCTAT	This study
RT-1987-R	AATATCCGCAGCTCATACTT	This study
RT-2107-F	GTCTTTGCCAACCTTTCC	This study
RT-2107-R	GCCATATCGTCATCTACTCT	This study
RT-*flhB*-F	TAAGCAGGAGATTAAGGATGAG	This study
RT-*flhB*-R	TAGTGGGTCGGGTTAGTC	This study
RT-*flhA*-F	ATCCTGATGAACGGCTATAC	This study
RT-*flhA*-R	GATACCGATGGCGAAGTT	This study
RT-*flhE*-F	GACTACTCTGTCTGGCTAT	This study
RT-*flhE*-R	GGGCTCAATAGGGTTATCT	This study
RT-*flgA*-F	CGATGGCTTCAATGTCAG	This study
RT-*flgA*-R	CTAGAGAACCAGCGTCAG	This study
RT-*flgB*-F	CGCTTTCACTCACCTCAT	This study
RT-*flgB*-R	ATAACGCAGGCTGTTGTC	This study
RT-*flhD*-F	TTGTTATTAGCACAGCGATT	This study
RT-*flhD*-R	CTGGCATACTAACTGATTGG	This study
RT-*fliC*1-F	TACTGGCGTTGATACTACC	This study
RT-*fliC*1-R	CTGAATAACATAGGCGGAAG	This study
RT-*fliC*2-F	CTTCACCGCCAATATCAAC	This study
RT-*fliC*2-R	GAGTTAGAGCCGTTCTGT	This study
RT-*fliA*-F	GAAGGCGTGATTGACAAA	This study
RT-*fliA*-R	TGAATGGCATAGGTGGTAA	This study
RT-*fliZ*-F	GAGTATGTGGTGCGTCTG	This study
RT-*fliZ*-R	CGAACTGAAGATACTGCTGATA	This study
RT-*fliD*-F	ACCACCAAGAGCATCAAT	This study
RT-*fliD*-R	CTTACTATTACTCATGGCGTTAA	This study
RT-*fliF*-F	CAGCCGCAATGAGAATATC	This study
RT-*fliF*-R	ACGCACCAGATTGTTGAT	This study
RT-*fliG*-F	CTGGAGGATATTCTGGAGTC	This study
RT-*fliG*-R	CAGGATAGTGGCGATGAT	This study
RT-*fliH*-F	GGGTTTTCAACAGGGACT	This study
RT-*fliH*-R	GGCAATGACGCTATCAAG	This study
RT-*fliI*-F	CATTAACGCTCTGCTGAC	This study
RT-*fliI*-R	CGCCGAGGATATTCTCAA	This study
RT-*fliJ*-F	AGATGCTACTCAACTATCAAGA	This study
RT-*fliJ*-R	CCAGCGTCAGGATAAACT	This study
RT-*fliK*-F	CCGTTTGCCGATATTCTG	This study
RT-*fliK*-R	CCGTGCTGATTCTCTAGG	This study
RT-*fliL*-F	AACGACTATCTGCCTGAG	This study
RT-*fliL*-R	TGAACGCTGTGAACATAAC	This study
RT-*fliM*-F	CAGCCGCAATGAGAATATC	This study
RT-*fliM*-R	ACGCACCAGATTGTTGAT	This study
RT-*fliN*-F	GTCAGAATAACGGCGATAT	This study
RT-*fliN*-R	GGATGTCCAGGATCAGAT	This study
RT-*fliO*-F	AGGTGGATAACCAATGTCT	This study
RT-*fliO*-R	CAGGAGTTCTCTTTACTTCG	This study
1902-OE-F	AGAAAAGAATTCAAAAGATCTAAAGAGGAGAAAGGATCTATGAAAAACCGTCAATTCATTTTACT	This study
1902-OE-R	GCCTGGAGATCCTTACTCGAGTCAGTTAAATTCGTAGTTGACGCC	This study
2842-OE-F	AGAAAAGAATTCAAAAGATCTAAAGAGGAGAAAGGATCTATGGATTCGCTGTCCATTCG	This study
2842-OE-R	GCCTGGAGATCCTTACTCGAGCTAAAGCGTTTTTACACAATTCGC	This study
*adrA*-OE-F	AGAAAAGAATTCAAAAGATCTAAAGAGGAGAAAATGTTCCCAAAA	This study
*adrA*-OE-R	GCCTGGAGATCCTTACTCGAGTCAGGCCGCCACTTCGGT	This study

## Data Availability

The data presented in this study are available in the article or Supplementary Materials.

## Supplementary Materials

The following supporting information can be downloaded at: https://www.mdpi.com/article/10.3390/pathogens11040464/s1, Figure S1: Alignment of the sequences of ThiB, ThiP, and ThiQ; Figure S2: The survival rates of turbot infected with ZW1 and ZW1ΔTT; Figure S3: The effect of TT deletion on bacterial motility; Figure S4: The effects of TT deletion on the expression of flagellar genes; Figure S5: Bacteria recovery from turbot peripheral blood leukocytes (PBLs) infected with Edwardsiella piscicida strains; Figure S6: The effects of TT deletion on the expression of adhesion factor genes; Figure S7: The effect of adrA overexpression on bacterial motility; Table S1: Bacterial strains used in this study.
