# Supplementary material for: The Involvement of Thiamine Uptake in the Virulence of Edwardsiella piscicida
Source: Pathogens. 2022 Apr 13;11(4):464. doi: 10.3390/pathogens11040464 (PMC9026889; doi:10.3390/pathogens11040464)
Supplement: Supplementary file 1 [file pathogens-11-00464-s001.zip › pathogens-1643447-supplementary.pdf]

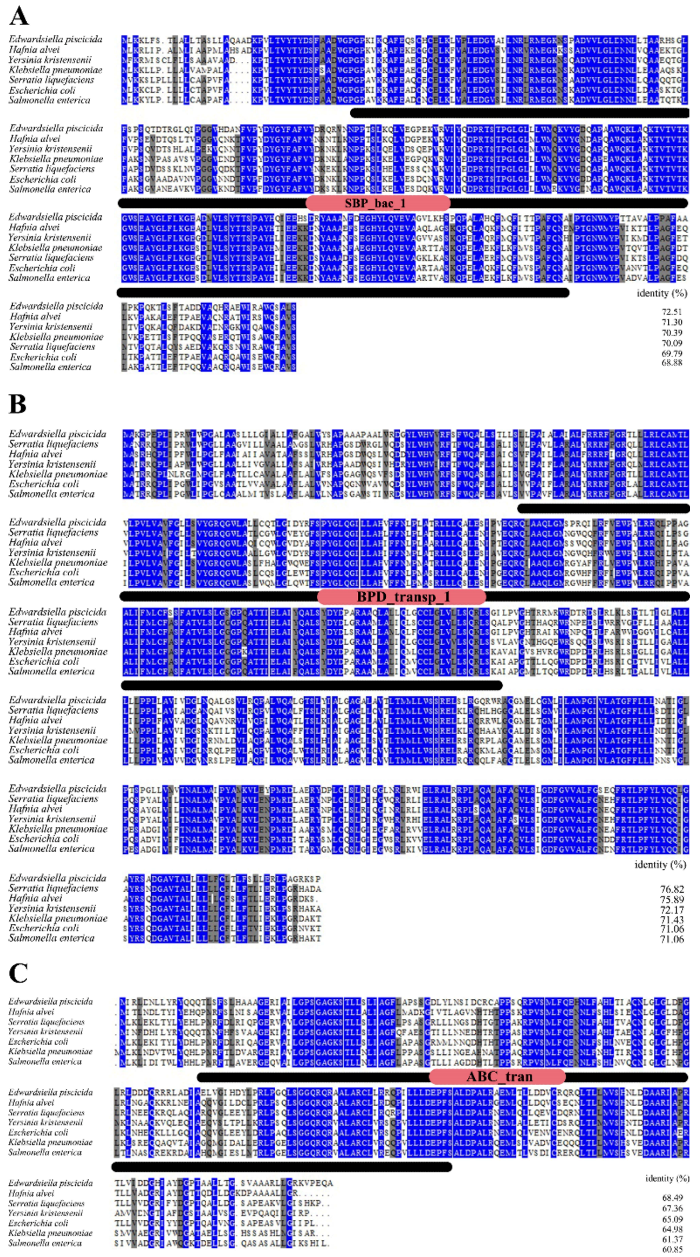

Figure S1. (**A**) Alignment of the sequences of ThiB, ThiP, and ThiQ. DNAMAN 9.0 was used to perform the multiple sequence alignment. (**B**) Protein domains were predicted by simple modular architecture research tool (SMART). (**C**) The consensus residues are in blue, the residues that are ≥75% identical among the aligned sequences are in gray. The sequence ID for ThiB are as follows: *Hafnia alvei*, WP_130987237.1; *Yersinia kristensenii*, WP_219654215.1; *Klebsiella pneumoniae*, VAX92858.1; *Serratia liquefaciens*, WP_116691298.1; *Escherichia coli*, CAD6022262.1; *Salmonella enterica*, CAD5307406.1. The sequence ID for ThiP are as follows: *S. liquefaciens*, HAU5996648.1; *H. alvei*, WP_004094607.1; *Y. kristensenii*, WP_219654216.1; *K. pneumoniae*, CDO16023.1; *E. coli*, CAD6017196.1; *S. enterica*, Q8ZRV1.1. The sequence ID for ThiQ are as follows: *H. alvei*, WP_046359690.1; *S. liquefaciens*, WP_116691363.1; *E. coli*, TFQ24710.1; *Y. kristensenii*, WP_219647317.1; *K. pneumoniae*, CCI75219.1; *S. enterica*, Q8ZRV2.1.

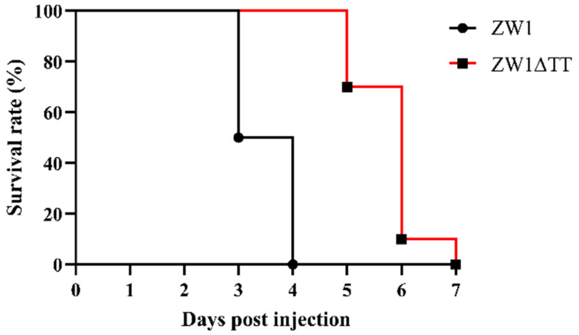

Figure S2. The survival rates of turbot infected with ZW1 and ZW1ΔTT. Turbot were injected intramuscularly with the wildtype *Edwardsiella piscicida* strain ZW1 or the TT mutant ZW1ΔTT at the same dose. The fish were monitored daily for survival

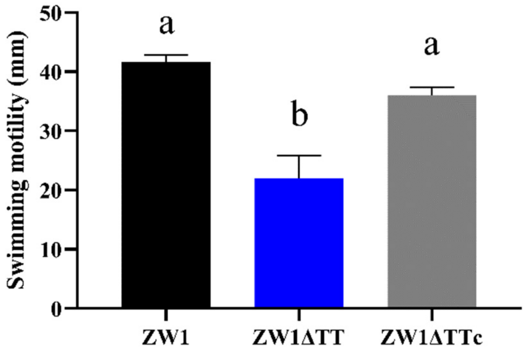

Figure S3. The effect of TT deletion on bacterial motility. ZW1, ZW1ΔTT, and ZW1ΔTTc were spotted onto the center of LB plates containing 0.3% agar. The plates were incubated at 28 °C for 48 h. The diameters of bacterial halos were recorded. Data are presented as means ± SD, $n = 3$. Data marked with different letters have statistically significant differences ($p < 0.05$).

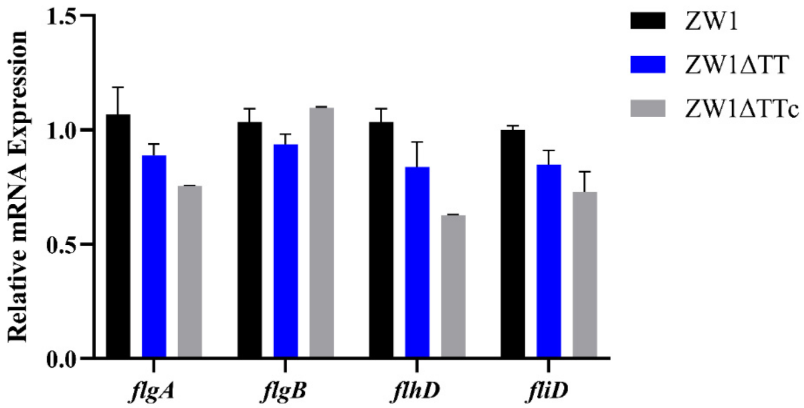

Figure S4. The effects of TT deletion on the expression of flagellar genes**.** The expression of *flgA*, *flgB*, *flhD*, and *fliD* in ZW1, ZW1ΔTT, and ZW1ΔTTc was determined by qRT-PCR. For the convenience of comparison, the expression levels in ZW1 were normalized as 1. Data are presented as means ± SD, $n = 3$.

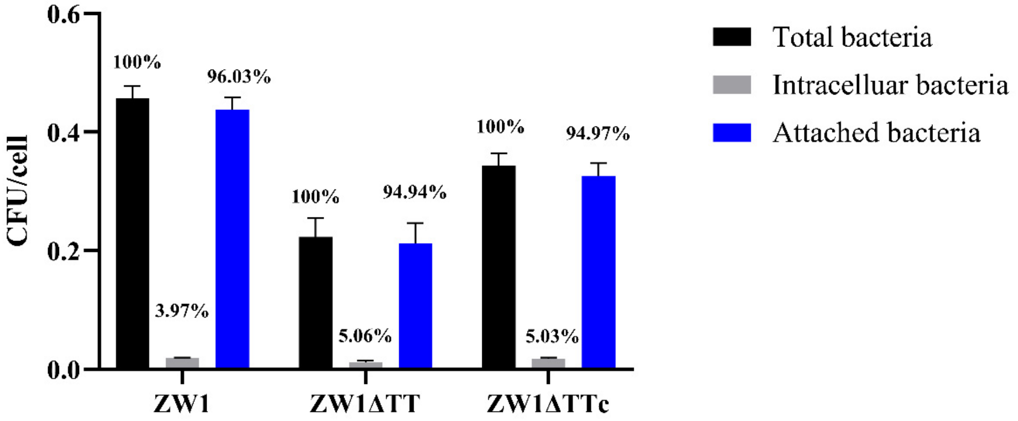

Figure S5. Bacteria recovery from turbot peripheral blood leukocytes (PBLs) infected with *Edwardsiella piscicida* strains. Turbot PBLs were infected with ZW1, ZW1ΔTT, or ZW1ΔTTc for 2 h at an MOI of 1:1. A portion of the cells was immediately lysed and determined for total bacterial recovery by plate count. Another portion of the cells was treated with gentamycin to kill the extracellular and surface-attached bacteria; the intracellular bacterial recovery was then determined by plate count. The amount of cell surface-attached bacteria was calculated by subtracting the intracellular bacterial recovery from the total bacterial recovery. For each strain, the total bacterial recovery was set as 100% for the convenience of comparison. Data are presented as means ± SD, $n = 3$.

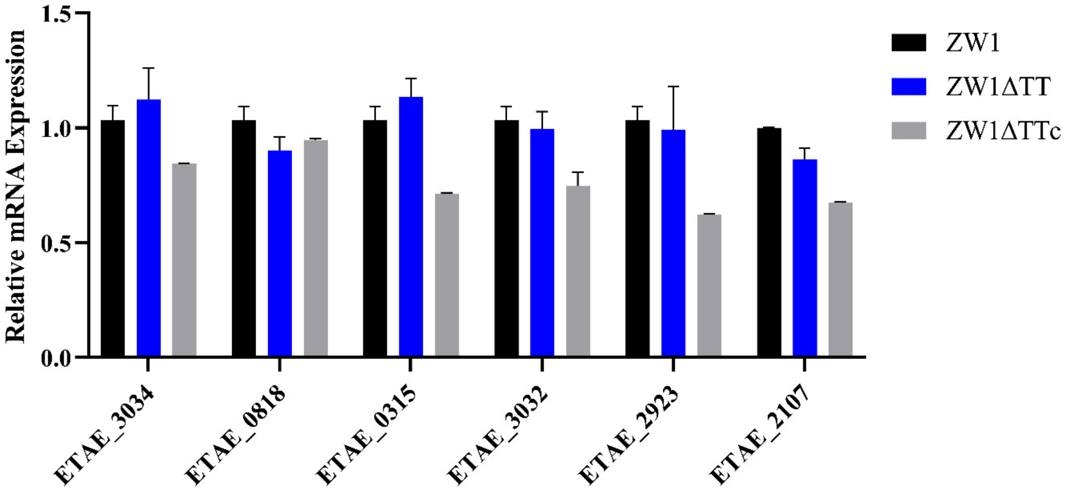

Figure S6. The effects of TT deletion on the expression of adhesion factor genes. The expression of ETAE_3034, ETAE_0818, ETAE_0315, ETAE_3032, ETAE_2923, and ETAE_2107 in ZW1, ZW1ΔTT, and ZW1ΔTTc was measured by qRT-PCR. For the convenience of comparison, the expression levels in ZW1 were normalized as 1. Data are presented as means ± SD, $n = 3$.

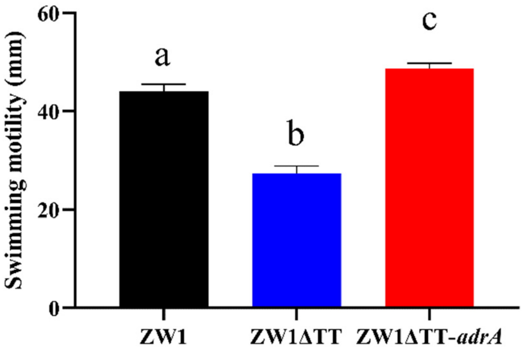

Figure S7. The effect of *adrA* overexpression on bacterial motility. ZW1, ZW1ΔTT, and ZW1ΔTT-*adrA* were spotted onto the center of LB plates containing 0.3% agar. The plates were incubated at 28 °C for 48 h. The diameters of bacterial halos were compared. Data are presented as means ± SD, *N*=3. Data marked with different letters have statistically significant differences ($p < 0.05$).

Table S1. Bacterial strains used in this study.

Bacterial strains	Description[1]	Source
Edwardsiella piscicida		
ZW1	Wild type strain, PMB^r	This study
ZW1ΔTT	ZW1, with the thiamine transporter operon knockout, PMB^r, Amp^r	This study
ZW1ΔTTc	ZW1ΔTT harboring the pCP1-TT, PMB^r, Amp^r	This study
ZW1/pCP1	ZW1 harboring the control plasmid pCP1, PMB^r, Amp^r	This study
ZW1ΔTT/pCP1	ZW1ΔTT harboring the control plasmid pCP1, PMB^r, Amp^r	This study
ZW1/pOE1c	ZW1 harboring the control plasmid pOE1c, PMB^r, Amp^r	This study
ZW1ΔTT/pOE1c	ZW1ΔTT harboring the control plasmid pOE1c, PMB^r, Amp^r	This study
ZW1ΔTT-1902	ZW1ΔTT harboring the plasmid pOE1-ETAE_1902, PMB^r, Amp^r	This study
ZW1ΔTT-2842	ZW1ΔTT harboring the plasmid pOE1-ETAE_2842, PMB^r, Amp^r	This study
ZW1/pOE1-*adrA**	ZW1 harboring the control plasmid pOE1-*adrA**, PMB^r, Amp^r	This study
ZW1ΔTT/pOE1-*adrA**	ZW1ΔTT harboring the control plasmid pOE1-*adrA**, PMB^r, Amp^r	This study
ZW1ΔTT-*adrA*	ZW1ΔTT harboring the plasmid pOE1-*adrA*, PMB^r, Amp^r	This study
ZW1/pEGFP-N2	ZW1 harboring the control plasmid pEGFP-N2, PMB^r, Kan^r	This study
ZW1ΔTT/pEGFP-N2	ZW1ΔTT harboring the control plasmid pEGFP-N2, PMB^r, Kan^r	This study

ZW1/pNmA	ZW1 harboring the plasmid pNmA, PMB^r, Kan^r	This study
ZW1ΔTT/pNmA	ZW1ΔTT harboring the plasmid pNmA, PMB^r, Kan^r	This study
ZW1/pBc3-5	ZW1 harboring the plasmid pBc3-5, PMB^r, Kan^r	This study
ZW1ΔTT/pBc3-5	ZW1ΔTT harboring the plasmid pBc3-5, PMB^r, Kan^r	This study
Escherichia coli		
DH5α	Cloning host	Tsingke, China
MG1655	Clone template of *adrA*	ATCC

[1] PMB, Polymyxin B; Amp, ampicillin; Kan, kanamycin. [*] Inactivated mutant.
